# Genome-Wide Characterization of Gibberellin Oxidase Genes (*GbGAoxs*) and Illustration of Their Molecular Responses to Exogenous GA_3_ in *Gossypium barbadense*

**DOI:** 10.3390/ijms26051985

**Published:** 2025-02-25

**Authors:** Zixin Zhou, Weiran Wang, Nan Zhao, Meng Wang, Jiahui Zhu, Jing Yang, Alifu Aierxi, Jie Kong

**Affiliations:** 1 Xinjiang Key Laboratory of Cotton Genetic Improvement and Intelligent Production, Cotton Research Institute of Xinjiang Uyghur Autonomous Region Academy of Agricultural Sciences, Urumqi 830000, China; zhouzixin2023@xaas.ac.cn (Z.Z.); wangweiran2011@xaas.ac.cn (W.W.); nan_zhao@cau.edu.cn (N.Z.); wangmeng2021@xaas.ac.cn (M.W.); zhujiahui2005@xaas.ac.cn (J.Z.); jingy2022@webmail.hzau.edu.cn (J.Y.); 2 National Cotton Engineering Technology Research Center, Xinjiang Uyghur Autonomous Region Academy of Agricultural Sciences, Urumqi 830000, China

**Keywords:** *Gossypium barbadense*, gibberellin oxidases, plant growth, gibberellin responses

## Abstract

As key enzymes in the gibberellin (GA) biosynthesis pathway, GAoxs function as regulators of bioactive GA levels and plant architecture, yet little is understood about GAoxs in *Gossypium*. In this study, 78 *GAox* genes identified in four cotton species were divided into three subgroups: GA2ox, GA3ox, and GA20ox. Syntenic relationships of GAoxs in *Gossypium* suggested that divergencies in gene function may be attributed to whole-genome duplication during evolution. *Cis*-acting element analysis suggested that the *GbGAox* genes might participate in plant growth, development, and hormone responses. Moreover, transcriptome analysis was performed to characterize the molecular response of the exogenous GA_3_ application. It was found that DEGs (differentially expressed genes) are widely involved in cell division and cell wall modification, in which the most *XTH* (xyloglucan endotransglucosylase/hydrolase) and *GAox* genes responded actively to the exogenous GA_3_ treatment. Some transcription factors and protein kinases cooperated with those GbGAoxs in response to GA_3_. These findings underlie the biological function of *GAox* genes and their responses to GA_3_ in regulating plant growth in *Gossypium barbadense*.

## 1. Introduction

Gibberellins (GAs) are necessary for diverse biological processes in plants, such as seed germination, stem elongation, leaf expansion, and anther and flower development [1,2,3,4]. Most of the gibberellin molecules have been identified as non-bioactive GAs that act as biosynthetic intermediates or inactivated catabolites. Among them, only GA_1_, GA_3_, GA_4_, and GA_7_ harbor biological activity [2,5]. The levels of bioactive GAs are regulated by the enzymes gibberellin–dioxygenases (GAox), which participate in the process of GA biosynthesis and deactivation [6].

Extensive studies of GA signaling pathways in plants have revealed that the biosynthesis and deactivation primarily consist of three stages. The first stage begins with the catalysis of geranylgeranyl diphosphate (GGPP) from *ent*-kaurene through the *ent*-copalyl diphosphate synthase (CPS) and *ent*-kaurene synthase (KS). The second stage involves the production of GA_12_ and GA_53_ according to *ent*-kaurene oxidase (KO) and *ent*-kaurenoic acid oxidase (KAO). The final stage involves the biosynthesis of GAs through the catalysis of GAoxs. The key enzymes GA20ox and GA3ox convert GA_12_ and GA_53_ into the bioactive GA_1_ and GA_4_, as well as some other GA intermediates. GA2oxs uniquely participate in the process of GA degradation, suppressing bioactive GAs and their precursors to maintain GA homeostasis [7]. The importance of GA balance in plant has led to increasing research exploring the mechanisms of GA signaling-pathway-related genes in plants [8].

Members of the *GAox* gene have been identified in a variety of plant species, like 16 in *Arabidopsis thaliana* [9], 24 in *Vitis vinifera* L. [10], and 13 in *Liriodendron chinense* [11]. In the *Rosaceae*, there are 140 in *Prunus avium* L., 146 in *P. mume*, and 113 in *Fragaria vesca* [12]. Studies have shown that GAoxs are involved in various processes. *TaGA20ox1* and *TaGA3ox2* are both involved in GA biosynthesis, which helps to break seed dormancy and to promote seed germination in wheat (*Triticum aestivum*) [13,14]. Notably, *GA2ox* genes are particularly functional in abiotic stress responses. Dwarf and delayed flowering 1 (*DDF1*) promotes the expression of *AtGA2ox7*, resulting in reduced endogenous GA biosynthesis and enhanced salinity tolerance [15]. Overexpression of *OsGA2ox8* enhances osmoprotectant accumulation, thereby improving osmotic stress tolerance in rice [16].

Cotton is an economically important crop producing textile fiber and cottonseed oil [17]. Studies have explored the function of phytohormones in the regulation of plant architecture, and GAs are critical for regulating stem elongation and tiller/branch number [18]. In the present study, *GAox* gene families in four cotton species are identified and systematically analyzed. Simultaneously, RNA-seq analysis is performed to explore the alterations in the expression of *GbGAox* genes and their associated signaling networks after the application of exogenous GA_3_. This research expands our understanding of the roles of gibberellin oxidase genes and provides potential candidates for molecular breeding in cotton.

## 2. Results

### 2.1. Identification and Evolutionary Analysis of GAox Genes in Cotton

**Figure 1:**
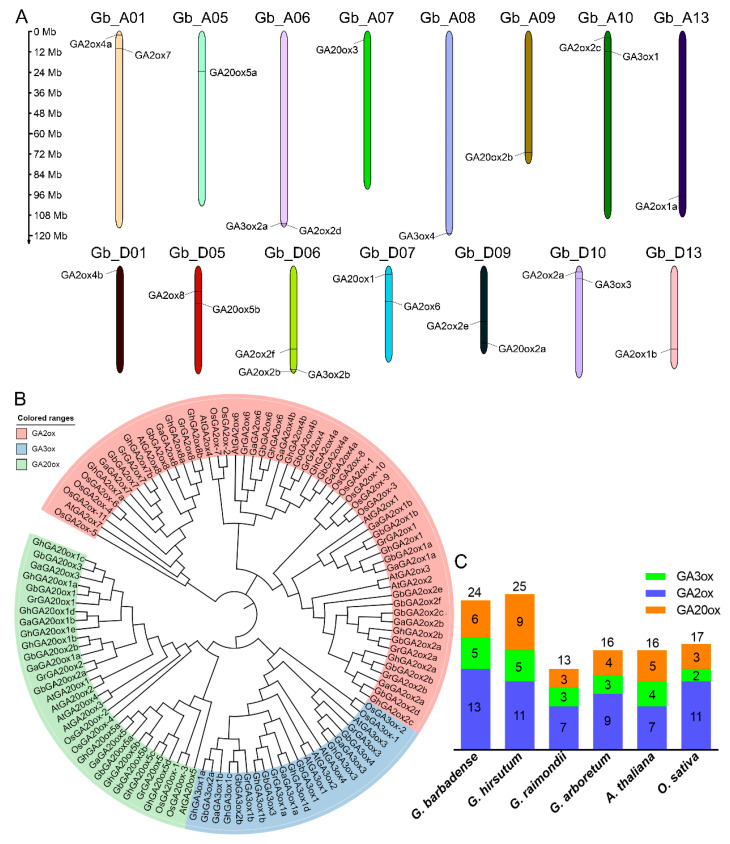
Chromosomal distribution and evolutionary analysis of *GbGAox* genes. (**A**) Illustrative diagram of the chromosomal distribution of *GbGAox* genes. Chromosome numbers are shown at the top of the bar. *GAox* genes are labeled on the chromosomes. The scale bar on the left indicates the chromosome lengths (Mb). (**B**) Phylogenetic analysis of *GAox* genes in *G. barbadense* (Gb), *G. hirsutum* (Gh), *G. arboretum* (Ga), *G. raimondii* (Gr), *A. thaliana* (At), and *O. sativa* (Os). The amino acid sequences were aligned with ClustalX, and the phylogenetic tree was constructed using the maximum likelihood (ML) method with 1000 bootstrap replicates. (**C**) Statistical results for the GAox family numbers in accordance with (**B**).

Combing the BLASTP search with 16 GAox sequences from Arabidopsis and Pfam analysis, a total of 24, 25, 16, and 13 *GAox* genes were predicted in *G. barbadense*, *G. hirsutum*, *G. arboretum*, and *G. raimondii*, respectively. The physicochemical parameters of the GAox proteins in the four cotton species are listed in Table S1. The *GbGAox* genes were unevenly distributed on 15 chromosomes, of which 11 were located in the At sub-genome and 13 in the Dt sub-genome (Figure 1A). Chromosome D06 contained the most *GbGAoxs* (3), whereas A05, A07, A08, A09, A13, D01, and D13 contained the fewest (1). To understand the phylogenetic relationship, GAox proteins from four cotton species and model plants like *Arabidopsis* and rice were carried out for evolutionary analysis. Based on the classification in *Arabidopsis*, three subgroups of *GAox* genes were identified in the selected six species, including *GA20ox*, *GA3ox*, and *GA2ox*. The number of *GA2oxs* was higher than that of *GA3oxs* and *GA20oxs* (Figure 1B,C), indicating that *GA2ox* genes have undergone a more dynamic evolutionary history compared to *GA3oxs* and *GA20oxs*, resulting in greater functional redundancy.

**Figure 2:**
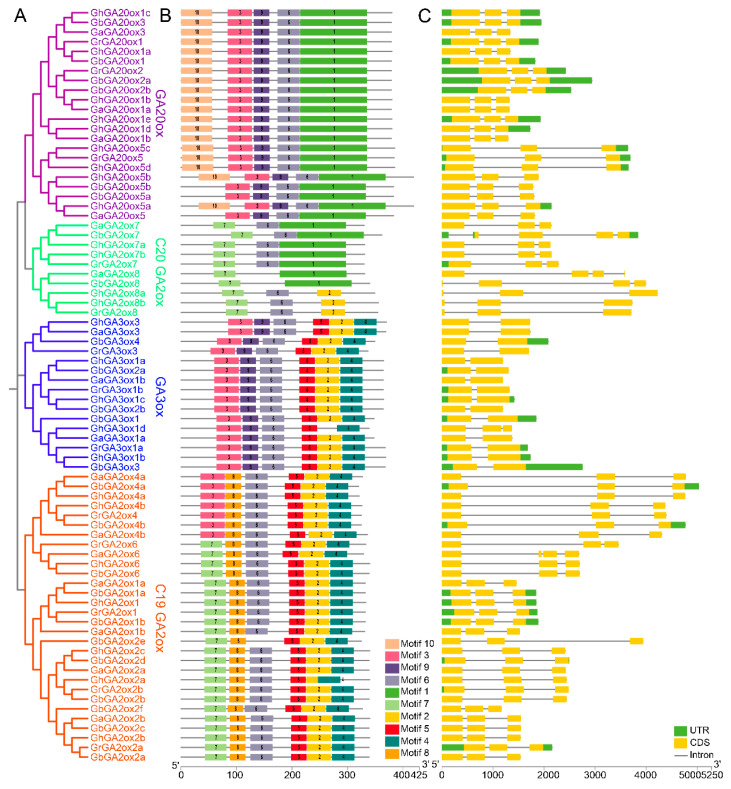
Phylogenetic relationship (**A**), conserved motifs (**B**), and gene structures (**C**) of GAox proteins in four cotton species. In (**A**), the phylogenetic tree was constructed using the MEGA 12 software based on the complete protein sequences of a total of 78 cotton GAox proteins. The subfamilies GA20ox, C20GA2ox, GA3ox, and C19GA2ox were labeled by purple, green, blue, and orange, respectively. In (**B**), the sequence logo for each motif is shown in Figure S1. In (**C**), exons and introns are shown by yellow boxes and black lines, respectively. UTRs are denoted by filled green boxes.

GAox proteins in four cotton species were further analyzed for structural and evolutionary features. Among the 78 proteins, a total of 10 GAox (two GbGAox, two GrGAox, four GhGAox, and two GaGAox) belonged to the C20-GA2ox subfamily, 30 GAox (11 GbGAox, five GrGAox, seven GhGAox, and seven GaGAox) belonged to C19-GA2ox (Figure 2A). The GAox proteins clustered within the same subfamilies had similar motif distributions and compositions, implying that these members had a similar evolutionary history (Figure 2B and Figure S1). Most GAox proteins had the conserved motif 6, except for GbGA2ox2e, GbGA2ox8, and GaGA2ox8. Interestingly, motif 10 was shared only in the GA20ox subfamily, implying that it may play a special function. Variations in motif composition and distribution may explain the functional differences in the evolutionary development of the *GAox* gene family. The exon–intron structure of each *GAox* gene found that the lengths and compositions of coding sequences were various, with most *GAox* genes containing three exons, whereas only two exons existed in the GA3ox subfamily (Figure 2C).

### 2.2. Duplication and Synteny Analyses of GbGAox Genes

**Figure 3:**
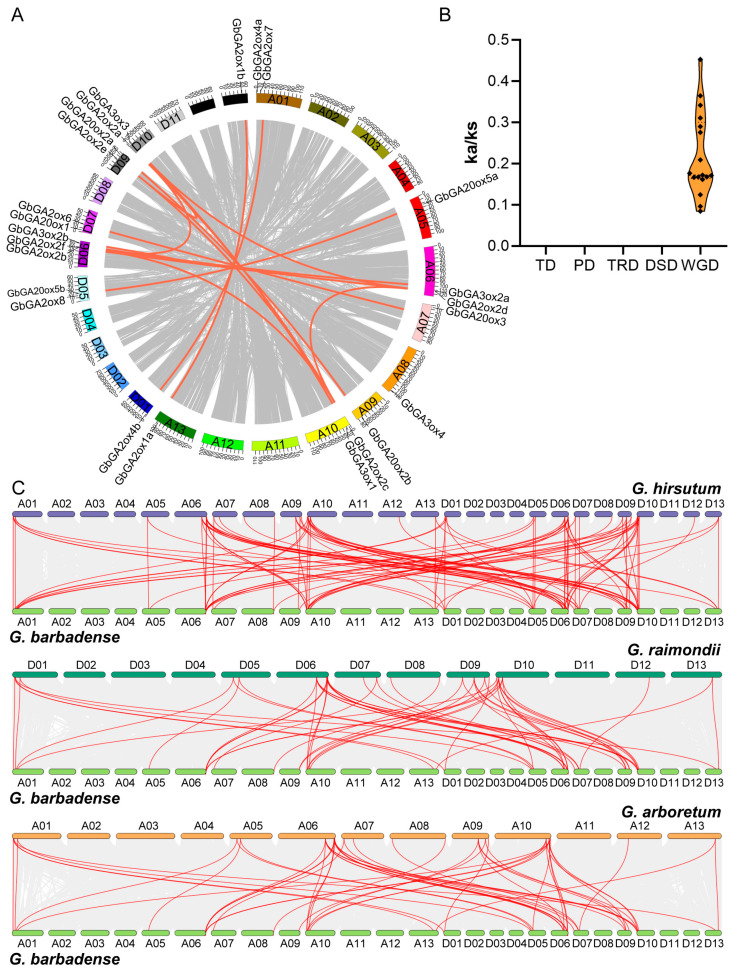
Gene duplication and synteny analysis of *GAox* genes. (**A**) Synteny analysis of the *GbGAox* gene family. The gray line in the background indicates a collinear block in the genome of *G. barbadense*, while the red line links duplicated gene pairs. (**B**) Comparison of the *Ka/Ks* values of *GbGAox* genes for different patterns of gene duplication. WGD, whole-genome duplication; PD, proximal duplication; TRD, transposed duplication; TD, tandem duplication; DSD, dispersed duplication. (**C**) Synteny analysis of *GAox* genes between *G. barbadense* and *G. hirsutum*/*G. raimondii*/*G. arboretum*, respectively. Red lines highlight the duplicated *GAox* gene pairs, and gray lines in the background indicate the collinear blocks.

Among the 24 *GbGAox* genes, there were 17 orthologous gene pairs identified in the sea island cotton genome (Figure 3A). In order to gain insights into the evolutionary constraints acting on the *GbGAox* gene family, the nonsynonymous (*Ka*)/synonymous (*Ks*) ratios of the gene pairs were calculated (Table S2). It is worth noting that the *Ka*/*Ks* ratios of all the identified 17 pairs were <1, and these genes were identified to be whole-genome duplications (Figure 3B), indicating that the *GbGAox* gene family had undergone strong purifying selection with limited functional divergence that occurred after whole-genome duplications (WGD). To further investigate the genome synteny in *Gossypium*, a comparative synteny map of *G. barbadense* was constructed in association with three other cotton genomes. A total of 54, 52, and 103 collinear pairs of homologous genes were detected between *GbGAoxs* and *GrGAoxs/GaGAoxs/GhGAoxs*, respectively (Figure 3C, Table S3), indicating a closer evolutionary relationship between sea island cotton and land cotton, compared to other cotton species.

### 2.3. Analyses of Cis-Acting Elements and Interaction Networks for GbGAox Genes

**Figure 4:**
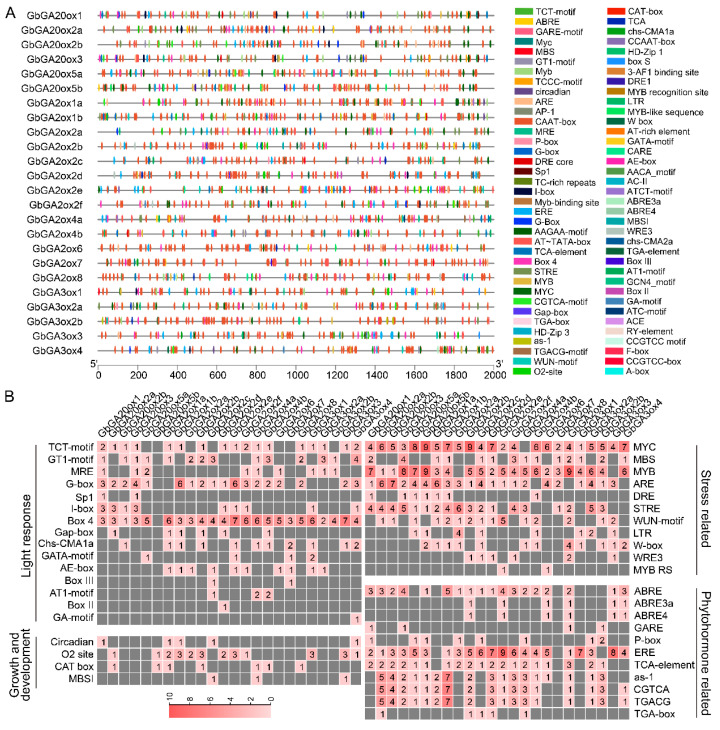
Analyses of *cis*-acting elements in the promoter regions of *GbGAox* genes. (**A**) Predicted *cis*-elements in the promoter regions of *GbGAox* genes. (**B**) Predicted *cis*-elements classified by function. In (**A**), the lower scale bar indicates the length of the promoter sequence. In (**B**), the different colors and numbers on the grid indicate the numbers of different *cis*-acting elements in each *GAox* gene.

Further analyses of the *GAox* genes were conducted by predicting the *cis*-elements within their promoter regions (Figure 4A). Plenty of regulatory elements existed and mainly involved the light response (GT1-motif, G-Box, Box4, TCT-motif, etc.), phytohormone response [gibberellin response (P-box, GARE-motif), abscisic acid response (ABRE), MeJA-response (CGTCA-motif, TGACG-motif), SA response (TCA-element, as-1) and so on], growth and development response (O2-site, circadian, etc.), and response to stress [drought-related (MBS, DRE), low-temperature response (LTR), etc.] (Figure 4B). A large number of *GAox* genes contained ABA, ER, SA, and MeJA responsive elements, illustrating the ability of *GbGAox* genes to respond to a variety of phytohormones.

### 2.4. Effect of Exogenous GA_3_ on Regulating Plant Growth in G. barbadense

**Figure 5:**
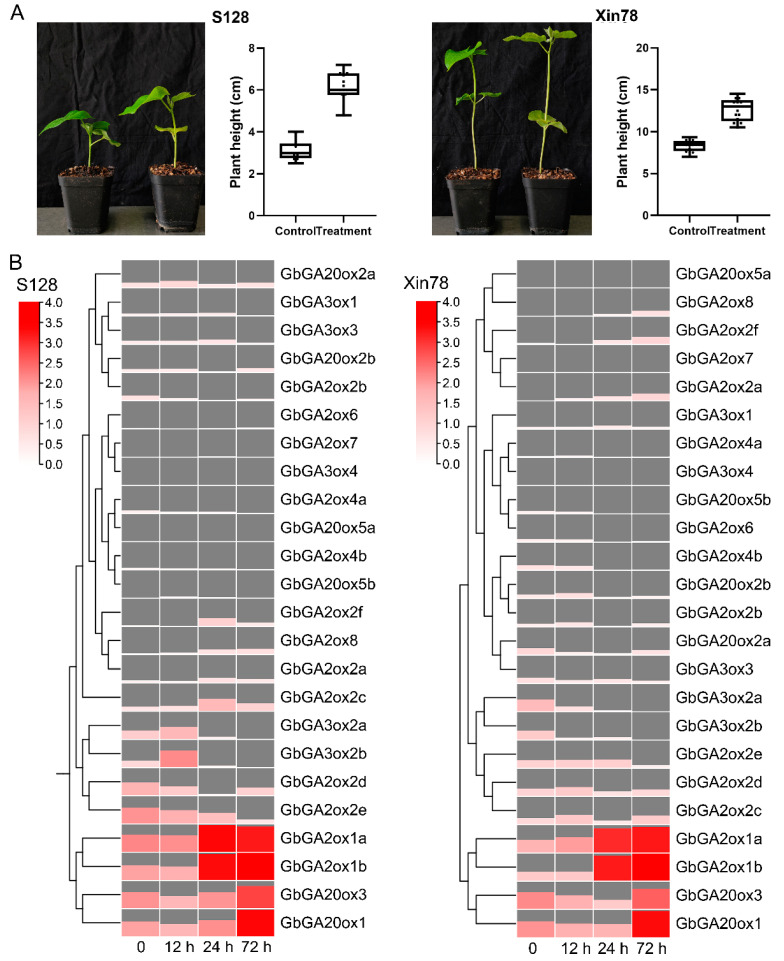
Expression profiles of *GbGAox* genes under the GA_3_ treatment in two sea island cultivars. (**A**) Phenotypic observation and changes in plant height after GA_3_ treatment in S128 and Xin78. (**B**) The variation trend of relative expression of *GbGAox* genes under the GA_3_ treatment. Non-treated (0 h) plants act as control. DEGs with standard by Log_2_(FPKM + 1). The redder the color, the higher the gene expression level. The left panel was clustered by gene expression level. The hours 12, 24, 72 h represent the treatment time of GA_3_. The height of the block and the color scale of the heatmap indicate the level of gene expression, with higher heights and redder colors indicating a higher level of gene expression.

To explore the effects of the exogenous GA_3_ treatment, two main cultivars of sea island cotton in Xinjiang (S128 and Xin78) were selected for treatment with 20 mg/L GA_3_. As shown in Figure 5A, after a two-week treatment of GA_3_, the plant height was significantly enhanced in both materials. According to the transcriptome data, similar increasing trends in *GA2ox1a*, *GA2ox1b*, *GA20ox1*, and *GA20ox3* along with the GA_3_ treatment were observed in both cultivars (Figure 5B), indicating that these genes may actively respond to a GA_3_ stimulus. In particular, *GA2ox1a* and *GA2ox1b*, and *GA3ox2a* and *GA3ox2b*, in both cultivars, showed similar patterns in the GA_3_ response, implying that their roles may be redundant.

### 2.5. Identification of DEGs and Functional Enrichment

**Figure 6:**
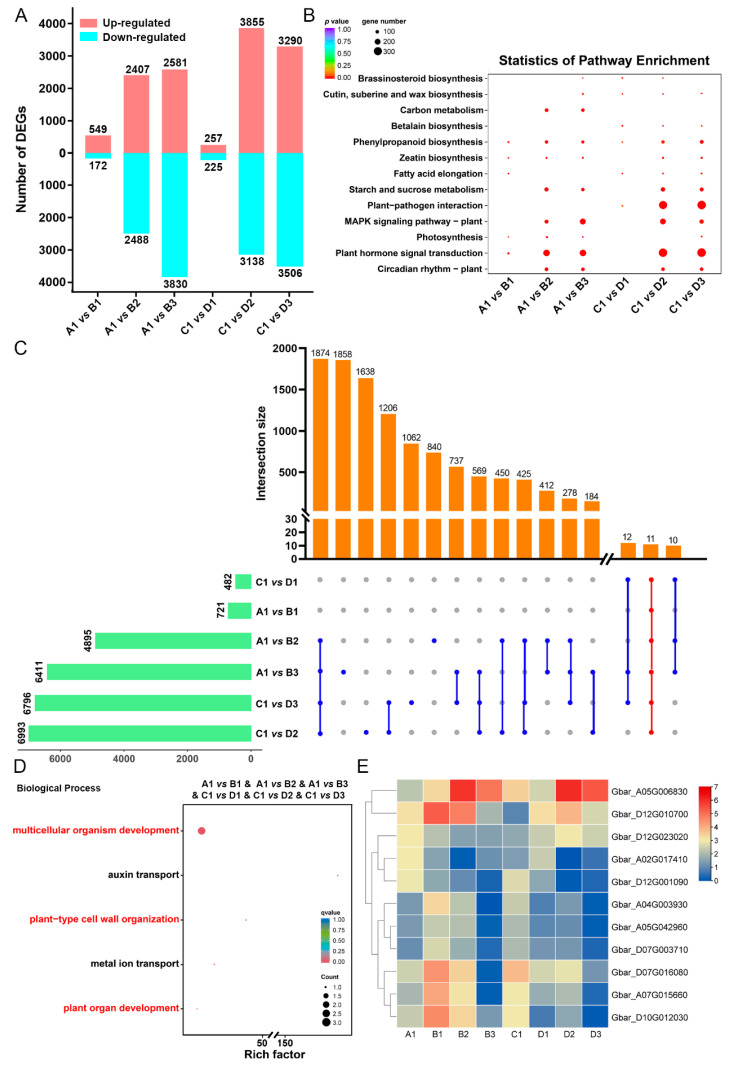
Comparison of DEGs in six pairs of two cotton cultivars treated with GA_3_. (**A**) The bar graph shows the number of up- and downregulated DEGs for different groups. (**B**) KEGG pathway enrichment analysis in different groups. (**C**) The bar graph shows partially overlapped and unique DEGs identified in two cotton cultivars treated with GA_3_. (**D**) GO (gene ontology) annotations for the overlapping DEGs in all comparisons. (**E**) The expression profile of 11 overlapping DEGs. A1, C1:S128, and Xin78 under normal conditions; B1–B3, D1–D3:S128 (**B**) and Xin78 (**D**) under the GA_3_ treatment for 12 h, 24 h, and 72 h, respectively. In (**D**), the rich factor represents the degree of enrichment, with larger values indicating greater enrichment. The size of the circle represents the number of genes, with bigger circles indicating more genes. The color of the circle represents the *p* value, with darker colors indicating smaller *p* values with higher significant differences.

Comparisons were conducted between the control (0 h) and the 20 mg/L GA_3_ treatments with different treatment times (12, 24, 72 h) in two cotton cultivars, respectively. Of those, more DEGs were identified under the long-time GA_3_ treatment (A1 vs. B2, A1 vs. B3, C1 vs. D2, C1 vs. D3) than the short-time treatment (A1 vs. B1, C1 vs. D1) (Figure 6A). KEGG (Kyoto Encyclopedia of Genes and Genomes) analyses revealed that DEGs were highly enriched in hormone production and signaling pathways, as well as substance metabolism (Figure 6B). The DEGs from six comparisons were selected for further investigation of the transcriptional changes induced by the GA_3_ treatment (Figure 6C). We noticed that 11 overlapped DEGs among all comparisons [A1 vs. B1, A1 vs. B2, A1 vs. B3, C1 vs. D1, C1 vs. D2, C1 vs. D3] were enriched in ‘multicellular organism development’, ‘auxin transport’, ‘plant-type cell wall organization’, ‘metal ion transport’, and ‘plant organ development’ process, all of which were widely involved in plant organogenesis (Figure 6D,E).

### 2.6. Gene Expression Patterns and Functional Enrichment of DEGs Under the GA_3_ Treatment

**Figure 7:**
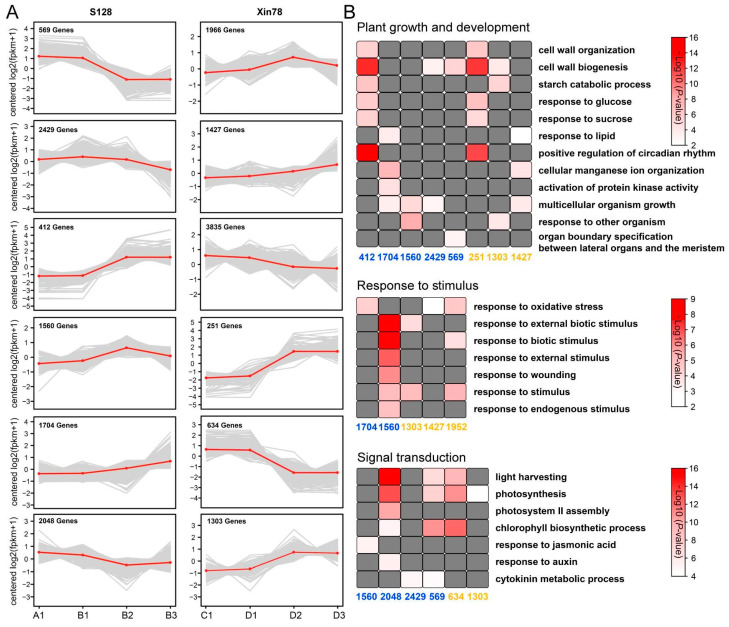
Changes in the expression profiles of DEGs in *G. barbadense* treated with GA_3._ (**A**) *K*-means clustering of the expression profiles of DEGs. (**B**) GO enrichment of functional categories among the six clusters. Clusters in S128 are shown in blue font and clusters in Xin 78 in yellow font. The color of the rectangle represents the *p*-value, with darker colors indicating smaller *p*-values and higher significant differences.

To elucidate the changes in gene expression under the GA_3_ treatment, DEGs were classified into six groups by using *K*-means clustering in Xin78 and S128, respectively (Figure 7A). Further, GO classification was performed to assess the functional significance of the transcriptional changes within each group, with the results indicating that clusters were widely relevant to plant growth and development (cell wall organization, starch catabolic process, response to glucose, sucrose, lipid, etc.), stimulus response (oxidative stress, biotic stimulus, wounding), and signal transduction (photosynthesis and hormone biosynthesis) (Figure 7B). Notably, the cluster 412 in S128 and the cluster 251 in Xin 78 were significantly enriched in plant organogenesis processes, and the overlaps between the two clusters (196 DEGs) were enriched in the process related to cell wall synthesis (Figure S2A,B).

### 2.7. Hormone Signal Transduction and Co-Expression of GAoxs

**Figure 8:**
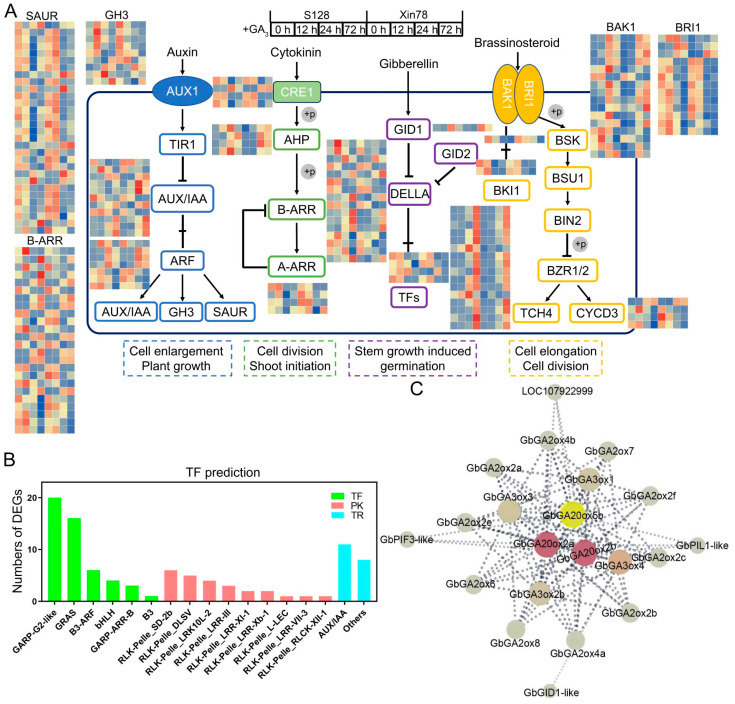
Interaction network of DEGs in response to the GA_3_ treatment. (**A**) Transcriptional changes in DEGs within the plant hormone signal transduction pathway after treatment with GA_3_. The grey circles containing ‘+P’ indicate phosphorylation events. The selected DEGs were those overlapping between X78 and S128, the heatmaps of the DEGs were generated based on the FPKM values normalized by the Z-score method. (**B**) TF prediction of DEGs in (**A**). TF: transcription factor; PK: protein kinase; TR: transcription regulator. (**C**) Co-expression network of *GbGAoxs* and DEGs related to the gibberellin pathway. The larger central circle represents a higher degree. The dashed lines indicate the predicted functional associations among different genes.

To further reveal the function of plant hormone signal transduction (ko04075) in relation to affecting the plant-type structure, DEGs (overlaps between S128 and Xin78) related to auxin, cytokinin, gibberellin, and brassinosteroid metabolism pathways were screened, further emerging as being involved in plant growth and cell division (Figure 8A). TF prediction showed that some transcription factors (TFs) and protein kinases (PKs) involved in the process of GA_3_ response, such as GARP (Golden2, ARR-B, Psr1), GRAS [gibberellic acid-insensitive (GAI), repressor of GA1-3 mutant (RGA), and scarecrow (SCR)], B3 (B3 DNA-binding domain), and RLK-Pelle (receptor-like kinase-Pelle) (Figure 8B). In particular, the gibberellin pathway, where the gibberellin insensitive dwarf1 gene *GID1* was significantly upregulated after treatment with GA_3_, was significantly downregulated at the same time as TF, which participates in the GA signal transduction. Furthermore, the protein–protein interaction network illustrated the relationship between *GbGAox* genes and DEGs involved in the gibberellin pathway. As shown in Figure 8C, three *GbGA20ox* genes function as the hub genes, four *GbGA3oxs* and ten *GbGA2oxs* participate in the signaling network, where three TFs, i.e., *GbGID*, *GbPIF* (phytochrome interacting factor), and *GbPIL* (phytochrome interacting bHLH factor-like), are predicted to cooperate with *GbGAoxs* in response to GA_3_.

**Figure 9:**
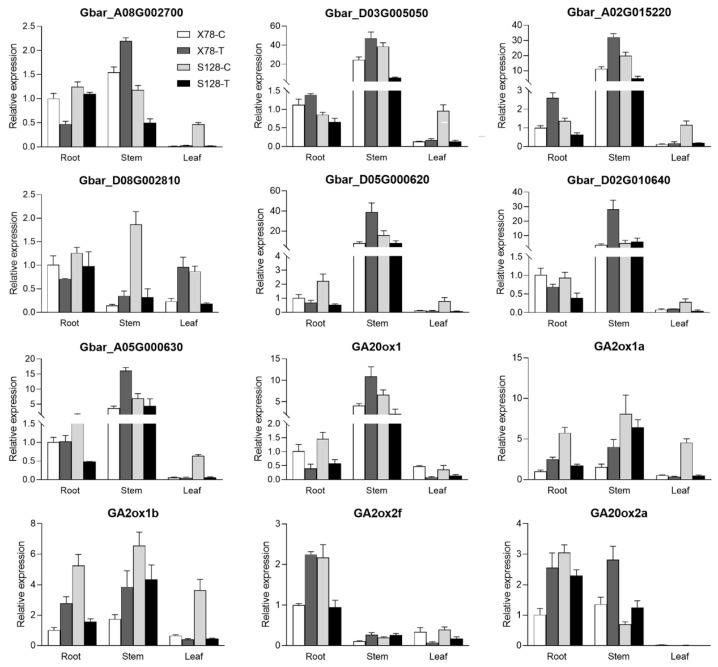
Changes in transcript profile of DEGs were validated by qRT-PCR. Tissues (root, stem, or leaf) were collected for measuring the expression profile of each gene under control and under the GA_3_ treatment for two weeks. C: control; T: treatment. The *GbUBQ7* gene was used as an internal control.

To further illustrate the expressional changes in *GbGAox* genes and DEGs involved in cell wall organization (Figure S2), seven DEGs and five *GAoxs* genes that were randomly selected were analyzed by quantitative RT-PCR. Two cotton cultivars were selected to perform the gene expression quantification analysis in different tissues treated with GA_3_. As shown in Figure 9, most selected genes were abundantly expressed in the stem, especially DEGs related to cell wall organization which were annotated as XTH. *GA20ox1* also presented a significantly higher expression profile in the stem and may be involved in stem elongation. However, most of these genes showed different expression trends in different materials, being upregulated in X78 but downregulated in S128 after the GA_3_ treatment.

## 3. Discussion

Bioactive GAs control diverse aspects of growth and development through complex metabolic and synthetic pathways [5]. The Green Revolution has revealed that the semi-dwarf wheat and rice are cultivated by manipulating key enzymes in the GA biosynthesis and metabolism which contribute to a substantial increase in crop yield [19,20]. Considering the great significance of gibberellin oxidase in the GA pathway, a comprehensive genome-wide evolutionary analysis of *GAox* genes was performed based on four cotton genomes. In this study, we systematically revealed information about these genes, including physicochemical properties, evolutionary events, and functional elements. Further, an exogenous GA_3_ treatment was performed to reveal the GA_3_ response of sea island cotton, as well as the pathway and genes involved in the response. Based on the above, the identification and functional analysis of the *GAox* gene family contribute to a better understanding of the GAoxs and GA pathways during plant growth in *G. barbadense*.

Here, a total of 78 *GAox* genes were identified from four cotton species, and the number of allotetraploids tested was approximately double that of diploids. The fewest number of *GAox* genes was present in *G. arboreum*, with only 13 members (Figure 1C). The synteny analysis revealed that the expansion of *GbGAox* genes was primarily driven by WGD events, consistent with the doubling of gene numbers in allotetraploid species compared to diploids (Figure 3B), meaning that functional redundancy may exist in the *GAox* gene family. The transcriptomic time course analysis revealed functional redundancy within *GAox* paralogs, exemplified by the synchronous upregulation of *GbGA2ox1a* and *GbGA2ox1b* following GA_3_ treatment (Figure 5B). *Cis*-acting element profiling further indicated that *GbGAox* promoters are enriched in light-, stress-, and phytohormone-related motifs (Figure 4B). Importantly, co-expression network analysis identified potential regulatory genes (e.g., *GbGID1* and *GbPIF3*) coordinating with key *GbGAox* members to participate in gibberellin signal transduction (Figure 8C).

Exogenous GAs have been used to study the function of GA in regulating plant-type structures. In watermelon, the loss function of *GA3ox* results in a dwarfism phenotype and an endogenous GA_4_ reduction, while it can be alleviated through application with GA_3_ or GA_4+7_, with the latter showing more significant effects [21]. Increasing evidence has revealed the effects of GA on root development, particularly the formation of lateral roots. Overexpression of *PcGA2ox1* causes GA deficiency in *Populus*, promoting lateral root proliferation and elongation, and these effects could be reversed with exogenous GA [22]. In addition, GA_3_ can promote fiber initiation and elongation [23]. In this study, plant height in two cotton varieties was promoted under the GA_3_ treatment, and the transcript levels of *GbGA2ox1a*, *GbGA20ox1b*, *GbGA20ox1*, and *GbGA20ox3* were significantly increased along with the GA_3_ treatment (Figure 5B), which may function as a regulator of the level of bioactive GAs.

To obtain the response pathway underlying the effects of GA_3_ on the regulation of plant growth, RNA-seq analyses were performed on two cotton cultivars under different GA_3_ treatment times. Analysis of GO and *K*-means cluster demonstrated that DEGs were significantly enriched in the progress of cell wall organization and plant organ development (Figure 6D and Figure 7B), and were annotated as *XTH* genes (Figure S2). As one of the key enzymes in plant cell wall remodeling, XTHs have been reported to be associated with the biosynthesis of the cell wall and the growth of cotton fibers [24,25]. In the hypocotyls of intact lettuce seedlings, GA_3_ strongly promotes elongation and increases the extractable XET (xyloglucan endotransglycosylase) activity [26]. Similarly, leaf elongation and higher levels of XET activity have been observed in the dwarf barley following GA_3_ treatment [27].

Moreover, PKs and TFs were involved in the response to the GA_3_ signal pathway (Figure 8). RLKs, such as SCRAMBLED/STRUBBELIG (SCM/SUB), have been reported to balance cell proliferation and differentiation [28]. LATETAL SUPPRESSORs (*LAS* in Arabidopsis, *LS* in tomato, *MOC1* in rice), belonging to GRAS TFs, have all been demonstrated to regulate axillary meristem initiation [29]. *GAox* genes have also been found to interact with bHLH TF (PIF, PIL) and GID TF, the former being reported to regulate photomorphogenic development and the latter acting as the GA receptor to regulate plant height [30,31]. In conclusion, *GbGAox* genes play crucial roles in GA biosynthesis, hormone response, and cell wall modification, and members of the XTH and GAox families actively respond to exogenous GA_3_ and coordinate with specific transcription factors and protein kinases to mediate the GA_3_ signaling pathway in *G. barbadense*.

## 4. Materials and Methods

### 4.1. Identification of GAox Genes in Four Gossypium Species

The genome sequences of *G. hirsutum*, *G. arboretum*, *G. raimondi*, and *G. barbadense* were downloaded from the Cottongen database (http://www.cottongen.org, accessed on 10 April 2024). The DIOX_N (PF14226) and 2OG-FeII_Oxy (PF03171) domains were used as a query to identify the *GAox* gene family in four cotton species. Then, the 16 *Arabidopsis* GAoxs (with seven AtGA2ox, four AtGA3ox, and five AtGA20ox) proteins were used for further authentication of GA20x, GA2ox, and GA3ox proteins of four *Gossypium* species, respectively. Protein sequences of AtGAoxs were downloaded from the TAIR database (https://www.arabidopsis.org, accessed on 10 April 2024). Physico-chemical properties of GbGAox proteins were obtained from ExPASy (https://www.expasy.org, accessed on 1 May 2024) [32]. Subcellular localizations of GbGAox proteins were predicted by using WoLF PSORT (https://wolfpsort.hgc.jp, accessed on 11 May 2024) [33].

### 4.2. Phylogeny, Gene Structure, and Conserved Motif Analyses of GAoxs

Phylogenetic relationships of GAox proteins among *Arabidopsis* [5], *Oryza sativa* [17], and four cotton species were aligned by ClustalX, and the phylogeny tree was constructed by MEGA 12. The Evolview online website was used to visualize the phylogenetic tree. Gene structures of GAoxs were analyzed and visualized by TBtools-II v2.142 [34]. All selected GAox proteins were submitted to the MEME Suite website (https://meme-suite.org/meme, accessed on 12 May 2024), and the number of motif sets was 10 [35].

### 4.3. Cis-Elements Analysis of GAox Genes

The 2000 bp upstream sequence from the transcription start site (TSS) for each *GbGAox* gene was submitted to the online website PLANTCARE (https://bioinformatics.psb.ugent.be/webtools/plantcare/html, accessed on 13 May 2024) for *cis*-element prediction [36].

### 4.4. Gene Duplication, Synteny Relationship, and Ka/Ks Analyses of GAox Genes

*GAox* genes were mapped to chromosomes according to the location information obtained from general feature format (gff) files of each cotton genome, and the positions were visualized through TBtools [34]. The GAox duplication events, types, and collinearity in the cotton genome were evaluated using multiple collinear scanning toolkits (MCScanX, https://github.com/wyp1125/MCScanX, accessed on 13 May 2024) [37]. The KaKs_Calculator 3.0 was used to calculate *Ka* and *Ks* rates of each duplicated *GAox* gene [38]. Circos (https://circos.ca/software/download/circos, accessed on 14 May 2024) was used to visualize location and homology relationships of *GAox* genes [39].

### 4.5. GA_3_ Treatments

There are two main cultivars (S128 and Xin78) of sea island cotton selected for GA_3_ treatment and transcriptome analysis. The plant height was determined as the length from the cotyledon node to the growth point of the main stem. Ten-day-old seedlings were treated with 20 mg/L of GA_3_, and phenotypic observations and trait measurements were performed after two weeks of treatment. Samples for RNA-seq were taken at 0, 12, 24, and 72 h after the administration of 20 mg/L of GA_3_ in approximately 1 cm sections below the stem apex.

### 4.6. RNA-Seq Analysis

Sequencing was performed on an Illumina HiSeq2000 Platform. The clean data were aligned with the sea island cotton genome reference (https://www.cottongen.org/species/Gossypium_barbadense/HAU-AD2_genome_v2.0, accessed on 20 February 2024) by HISAT2. FPKM (fragments per kilobase of transcript sequence per million mapped tags) values were used to quantify transcription level. DEGs (false discovery rate < 0.01 and fold change ≥ 2) were identified using the DEseq2 software (https://bioconductor.org/packages/release/bioc/html/DESeq2.html, accessed on 14 May 2024). STRING v12.0 and Cytoscape v3.9.1 were used to analyze and visualize protein–protein co-expression networks. Heatmaps were generated based on FPKM values normalized by the Z-score method, with colors indicating gene expression levels.

### 4.7. Quantitative Real-Time PCR Analysis

The total RNA was extracted from the tissues (roots, stems, and leaves) before and after the GA_3_ treatment of sea island cotton (S128 and Xin78) with the FastPure Universal Plant Total RNA Isolation Kit (Vazyme Biotech Co., Ltd., Nanjing, China). All the RNA samples (root, stem, leaf) were reversely transcribed to synthesize cDNA according to the instruction of HiScript II qRT SuperMix for qPCR (Vazyme Biotech Co., Ltd., Nanjing, China). qRT-PCR was performed using ChamQ Blue Universal SYBR qPCR Master Mix (Vazyme Biotech Co., Ltd., Nanjing, China) in a 7500 Real-Time PCR System (Applied Biosystems, Foster City, CA, USA), and all the primers used for qRT-PCR are shown in Table S4. PCR conductions were pre-denaturation at 95 °C for 30 s, denaturation at 95 °C for 10 s, annealing at 56 °C for 30 s, extension at 72 °C for 30 s, with a total of 40 cycles. Three biological replicates with two technical replicates were applied. The relative expression level of each gene was calculated by 2^−ΔΔCT^.

### 4.8. Statistical Analysis

Significant differences (*p* < 0.05) were investigated using t-tests or one-way ANOVA tests with Tukey’s multiple comparisons [40]. Data analysis was performed using IBM SPSS statistics 22 and the chart preparation was completed by GraphPad prism 8.

## 5. Conclusions

Overall, this is the first comprehensive and systematic genome-wide analysis of *GAox* genes in *Gossypium*. All these genes were further divided into four subgroups, and differences in motif composition and distribution among different subgroups could explain the functional divergence of the *GAox* gene family in the evolutionary history. The *GbGAox* gene family showed limited functional divergence due to whole-genome duplication, confirming a closer relationship between sea island cotton and upland cotton. In addition, transcriptome analysis found that DEGs were widely involved in cell division and cell wall modification when applied with exogenous GA_3_, and that GAoxs and their potential regulators may cooperate in regulating plant architecture through plant hormone signaling pathways. Our study contributes to the understanding of the potential role of GAoxs, providing elite candidate genes for plant architecture improvement.

## Data Availability

The original contributions presented in this study are included in the article/Supplementary Materials. Further inquiries can be directed to the corresponding author(s).

## Supplementary Materials

The following supporting information can be downloaded at: https://www.mdpi.com/article/10.3390/ijms26051985/s1.
