# Supplementary material for: Genome-Wide Characterization of Gibberellin Oxidase Genes (GbGAoxs) and Illustration of Their Molecular Responses to Exogenous GA3 in Gossypium barbadense
Source: Int J Mol Sci. 2025 Feb 25;26(5):1985. doi: 10.3390/ijms26051985 (PMC11899772; doi:10.3390/ijms26051985)
Supplement: Supplementary file 1 [file ijms-26-01985-s001.zip › ijms-3404173-supplementary.pdf]

Supplementary Materials Data

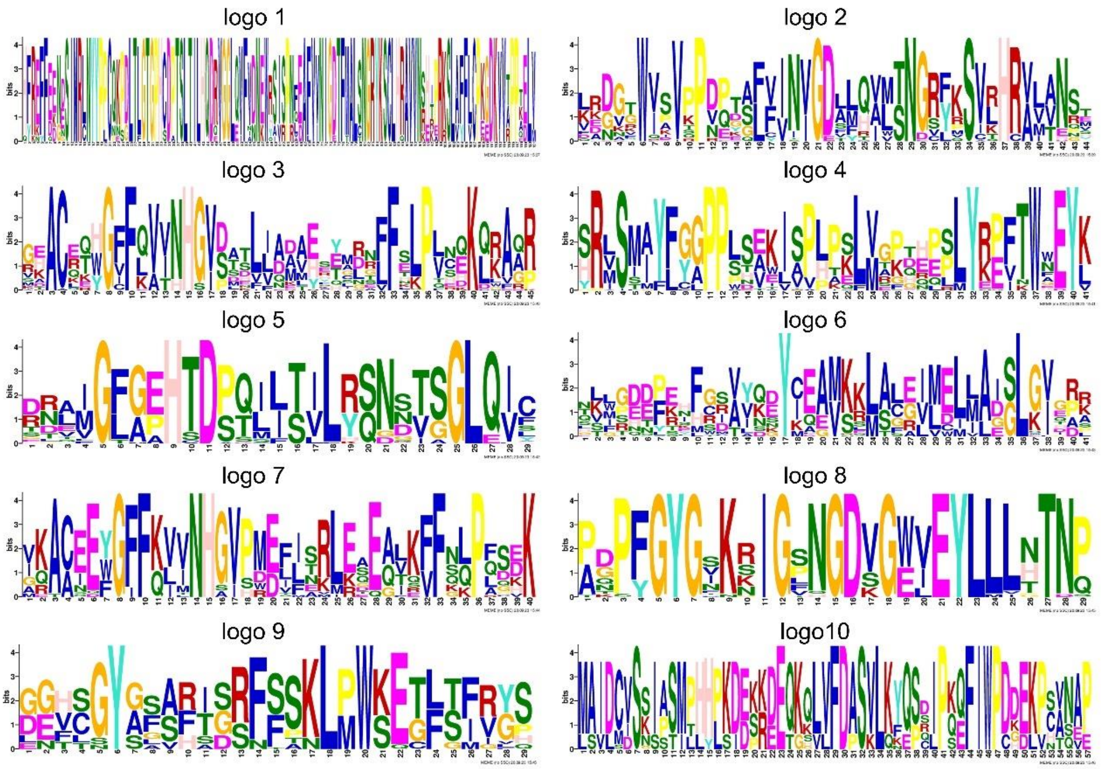

Figure S1. Conserved motifs of GAox proteins in cotton. DNA sequence motif represented as a sequence logo. Bits represents the degree of conservation of each position using the height of the consensus character at that position. The X-axis represents the length of the sequence.

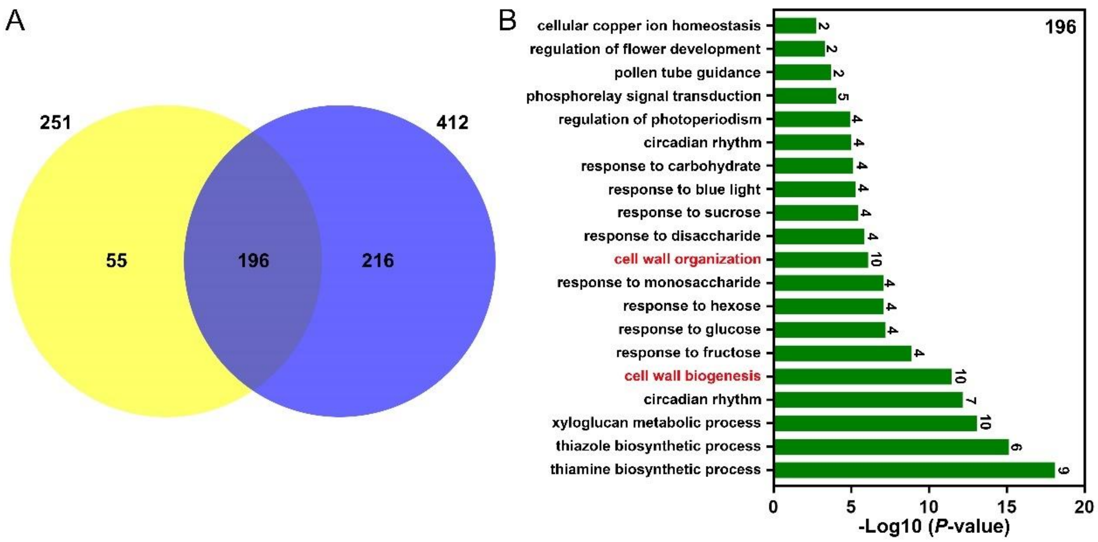

Figure S2. Venn analysis and GO enrichment of clusters involved in plant organogenesis from cultivar S128 and Xin78. (**A**) Venn analysis of cluster 251 in Xin 78 and cluster 412 in S128. (**B**) The most enriched TOP 20 GO terms of the overlapping DEGs.

Table S1. Basic information about the members of the *GAox* gene family in cotton.

Gene name	Gene ID	Chr	Start	End	AA	MW (kDa)	pI	Subcellular location
GaGA20ox1a	Ga09G1936	Chr09	77336565	77337890	379	43.17521	6.98	cyto
GaGA20ox1b	Ga09G0545	Chr09	29188235	29189537	379	43.27839	7.13	nucl/cyto
GaGA20ox3	Ga07G0488	Chr07	5293873	5295215	379	43.09311	6.56	nucl/cyto
GaGA20ox5	Ga05G2543	Chr05	24598972	24600792	383	43.62032	6.98	chlo
GaGA2ox1a	Ga13G1903	Chr13	110114395	110115858	332	37.33996	8.19	nucl
GaGA2ox1b	Ga01G0389	Chr01	3599563	3601089	331	37.16058	9.03	nucl/cyto
GaGA2ox2a	Ga06G2463	Chr06	130451279	130453703	339	38.19872	6.51	cyto/nucl
GaGA2ox2b	Ga10G2652	Chr10	125632715	125634262	340	38.0967	8.11	cyto/nucl/chlo
GaGA2ox4a	Ga01G0322	Chr01	2550103	2554878	327	35.79999	6.95	nucl/cyto
GaGA2ox4b	Ga09G2749	Chr09	84393505	84397811	336	37.21358	6.75	cyto
GaGA2ox6	Ga07G1447	Chr07	26873661	26876347	329	36.66991	7.86	nucl/cyto
GaGA2ox7	Ga01G0804	Chr01	12021871	12024013	330	37.90516	7.45	nucl/cyto
GaGA2ox8	Ga05G1796	Chr05	16354830	16358415	331	38.37184	7.99	nucl/cyto
GaGA3ox1a	Ga10G2260	Chr10	118538432	118539810	348	38.88232	7.8	nucl
GaGA3ox1b	Ga06G2297	Chr06	129049475	129050670	364	40.53133	7.96	chlo/nucl
GaGA3ox3	Ga08G2824	Chr08	128258597	128260329	369	42.19454	6.29	chlo/nucl
GbGA20ox1	Gbar_D07G004640	ChrD07	4889432	4891262	379	43.22227	6.57	cyto/nucl
GbGA20ox2a	Gbar_D09G018790	ChrD09	45248729	45251671	379	43.1892	6.88	cyto
GbGA20ox2b	Gbar_A09G018990	ChrA09	71224158	71226690	379	43.15419	6.98	cyto/nucl
GbGA20ox3	Gbar_A07G004340	ChrA07	5222281	5224230	379	43.10713	6.56	nucl/cyto
GbGA20ox5a	Gbar_A05G023670	ChrA05	23613903	23615712	383	43.61633	6.98	chlo
GbGA20ox5b	Gbar_D05G024240	ChrD05	21977586	21979373	383	43.68539	6.98	chlo
GbGA2ox1a	Gbar_A13G016640	ChrA13	96601350	96603197	332	37.33996	8.19	nucl
GbGA2ox1b	Gbar_D13G016610	ChrD13	48929497	48931387	332	37.35403	8.44	nucl
GbGA2ox2a	Gbar_D10G004310	ChrD10	3493092	3494634	339	37.96661	8.11	cyto/chlo
GbGA2ox2b	Gbar_D06G021870	ChrD06	61831569	61834013	339	38.24375	6.52	cyto/nucl
GbGA2ox2c	Gbar_A10G004440	ChrA10	3786959	3788506	339	38.0677	8.11	cyto/chlo/nucl
GbGA2ox2d	Gbar_A06G021100	ChrA06	114014099	114016593	339	38.18675	6.51	cyto/nucl
GbGA2ox2e	Gbar_D09G008430	ChrD09	32239436	32243378	325	36.31889	9.22	chlo/chlo_mito
GbGA2ox2f	Gbar_D06G015470	ChrD06	48693205	48694375	327	36.69036	7.31	cyto/nucl
GbGA2ox4a	Gbar_A01G002810	ChrA01	2556676	2561708	320	35.06616	6.85	nucl/cyto
GbGA2ox4b	Gbar_D01G002990	ChrD01	2579012	2583783	325	35.78296	6.74	nucl/cyto
GbGA2ox6	Gbar_D07G014160	ChrD07	20684186	20686884	339	37.67702	6.94	nucl/cyto
GbGA2ox7	Gbar_A01G006610	ChrA01	10591133	10594976	362	41.48414	7.84	nucl/cyto
GbGA2ox8	Gbar_D05G017240	ChrD05	14929410	14933405	340	39.51339	7.99	nucl/cyto

GbGA3ox1	Gbar_A10G008380	ChrA10	12180664	12182515	348	38.8924	7.8	nucl
GbGA3ox2a	Gbar_A06G020170	ChrA06	112957576	112958884	364	40.55835	7.96	chlo/nucl
GbGA3ox2b	Gbar_D06G020970	ChrD06	60815059	60816255	364	40.59134	7.67	nucl/chlo
GbGA3ox3	Gbar_D10G007440	ChrD10	7722276	7725032	368	40.79345	7.31	nucl
GbGA3ox4	Gbar_A08G026180	ChrA08	118668102	118670189	349	39.71258	5.8	chlo/cyto
Gh20ox1a	Gh_D07G045600	ChrD07	4856175	4857517	380	43.21326	6.51	nucl/cyto
Gh20ox1b	Gh_A09G196600	ChrA09	76577884	76579210	380	43.12717	6.98	cyto/nucl
Gh20ox1c	Gh_A07G044700	ChrA07	5284044	5285964	380	43.10713	6.56	nucl/cyto
Gh20ox1d	Gh_A09G047000	ChrA09	18533985	18535718	380	43.29445	7.12	nucl/cyto
Gh20ox1e	Gh_D09G051300	ChrD09	18233038	18234972	380	43.15817	6.83	nucl/cyto
Gh20ox5a	Gh_A05G223800	ChrA05	24511641	24513788	419	47.33777	6.97	extr/chlo/vacu
Gh20ox5b	Gh_D05G240000	ChrD05	22030390	22032284	419	47.42481	6.97	extr/chlo/vacu
Gh20ox5c	Gh_D09G004600	ChrD09	958836	962481	385	43.37213	6.57	nucl/cyto
Gh20ox5d	Gh_A09G005200	ChrA09	1119176	1122831	385	43.32001	6.37	nucl/cyto
Gh2ox1	Gh_A13G172500	ChrA13	96253304	96255155	333	37.33996	8.19	nucl
Gh2ox2a	Gh_D06G234500	ChrD06	64982868	64985312	340	38.24375	6.52	cyto/nucl
Gh2ox2b	Gh_D10G042100	ChrD10	3664057	3665600	340	37.99466	8.11	cyto/chlo
Gh2ox2c	Gh_A06G227200	ChrA06	124978378	124980797	340	38.18675	6.51	cyto/nucl
Gh2ox4a	Gh_A01G032000	ChrA01	2843428	2848191	321	35.06616	6.85	nucl/cyto
Gh2ox4b	Gh_D01G029400	ChrD01	2533437	2537811	326	35.80201	6.85	nucl/cyto
Gh2ox6	Gh_D07G138400	ChrD07	20711151	20713849	340	37.67702	6.94	nucl/cyto
Gh2ox7a	Gh_A01G076300	ChrA01	10828537	10830662	331	37.89113	7.45	nucl/cyto
Gh2ox7b	Gh_D01G071400	ChrD01	8986291	8988439	331	37.96324	7.45	cyto/nucl
Gh2ox8a	Gh_A10G006100	ChrA10	496878	501101	349	39.74929	7.11	nucl/cyto
Gh2ox8b	Gh_D10G016300	ChrD10	1294882	1298612	356	40.5804	6.17	extr/vacu/E.R plas
Gh3ox1a	Gh_A06G217500	ChrA06	123813242	123814438	365	40.55835	7.96	chlo/nucl
Gh3ox1b	Gh_D10G074400	ChrD10	8376554	8378291	369	40.73541	7.5	nucl
Gh3ox1c	Gh_D06G223900	ChrD06	63795931	63797353	365	40.56431	7.67	chlo/nucl/chlo_mito
Gh3ox1d	Gh_A10G066700	ChrA10	11710440	11711812	339	37.54882	7.07	nucl
Gh3ox3	Gh_A08G275600	ChrA08	124400538	124402266	370	42.1815	6.13	chlo
GrGA20ox1	Grai_07G024570	Chr07	48979028	48980922	379	43.33239	6.74	nucl/cyto
GrGA20ox2	Grai_09G021550	Chr09	44236391	44238818	379	43.19117	6.88	cyto/nucl
GrGA20ox5	Grai_09G000570	Chr09	1066742	1070434	384	43.35613	6.57	nucl/cyto
GrGA2ox1	Grai_13G020810	Chr13	47845439	47847309	332	37.35403	8.44	nucl
GrGA2ox2a	Grai_10G004350	Chr10	3258668	3260831	339	37.8975	7.83	cyto/chlo
GrGA2ox2b	Grai_06G026430	Chr06	59270387	59272869	339	38.24375	6.52	cyto/nucl
GrGA2ox4	Grai_01G003210	Chr01	2356472	2360866	325	35.78296	6.74	nucl/cyto
GrGA2ox6	Grai_08G004830	Chr08	4540964	4544424	333	37.09435	7.25	cyto/nucl
GrGA2ox7	Grai_01G007910	Chr01	8523188	8525474	330	37.92922	7.45	cyto/nucl

GrGA2ox8	Grai_10G001630	Chr10	1151402	1155112	355	40.5804	6.17	extr/vacu/E.R plas
GrGA3ox1a	Grai_10G007910	Chr10	7684094	7685777	368	40.8185	7.15	nucl
GrGA3ox1b	Grai_06G025490	Chr06	58398681	58400006	364	40.57838	7.96	nucl/chlo/chlo_mito
GrGA3ox3	Grai_08G031240	Chr08	61581506	61583211	337	38.24482	6.24	chlo/nucl

Chr: Chromosome; MW: Molecular weight of the amino acid sequence; pI: Theoretical isoelectric point. WoLF PSORT were used to predict the subcellular localization of the GAox family genes; the most likely locations are listed. nucl: nucleus; chlo: chloroplast; cyto: cytoplasmic; mito: mitochondrion; extr: extracell; vacu: vacuole; E.R plas: endoplasmic retuculum.

Table S2. Homologous gene pairs of the GbGAox gene family.

Seq_1	Seq_2	Ka	Ks	Ka_Ks
Gbar_A01G002810.1	Gbar_D01G002990.1	0.026514389	0.072784342	0.364286993
Gbar_A05G023670.1	Gbar_D05G024240.1	0.008527664	0.024986295	0.341293666
Gbar_A06G021100.1	Gbar_A10G004440.1	0.134507599	1.08151122	0.124370045
Gbar_A06G020170.1	Gbar_D06G020970.1	0.004841655	0.023152964	0.209115987
Gbar_A06G021100.1	Gbar_D06G021870.1	0.006357991	0.036056511	0.176334055
Gbar_A06G021100.1	Gbar_D06G015470.1	0.162224024	0.969819037	0.167272468
Gbar_A06G020170.1	Gbar_D10G007440.1	0.084152347	0.876699772	0.095987645
Gbar_A07G004340.1	Gbar_D07G004640.1	0.011380019	0.036654732	0.31046521
Gbar_A09G018990.1	Gbar_D09G018790.1	0.009160419	0.031625238	0.289655341
Gbar_A10G004440.1	Gbar_D06G015470.1	0.098484591	0.592342576	0.166262895
Gbar_A10G004440.1	Gbar_D09G008430.1	0.169505275	0.986145891	0.17188661
Gbar_A10G004440.1	Gbar_D10G004310.1	0.007730367	0.017082589	0.452529
Gbar_A10G008380.1	Gbar_D10G007440.1	0.011401043	0.041347017	0.275740397
Gbar_A13G016640.1	Gbar_D13G016610.1	0.004536631	0.053635901	0.084581982
Gbar_D06G015470.1	Gbar_D06G021870.1	0.162323331	0.949866831	0.170890619
Gbar_D06G015470.1	Gbar_D10G004310.1	0.09097796	0.563562067	0.16143379
Gbar_D09G008430.1	Gbar_D10G004310.1	0.162788185	0.969616297	0.167889283

Table S3. Orthologous relationships between *G. barbadense* and other three *Gossypium* species.

Gbar_A01	Gbar_A01G002810.1	==	A01	Gh_A01G032000.1
Gbar_A01	Gbar_A01G006610.1	==	A01	Gh_A01G076300.1
Gbar_A01	Gbar_A01G006610.1	==	A10	Gh_A10G006100.1
Gbar_A01	Gbar_A01G002810.1	==	D01	Gh_D01G029400.1
Gbar_A01	Gbar_A01G006610.1	==	D01	Gh_D01G071400.1
Gbar_A01	Gbar_A01G006610.1	==	D05	Gh_D05G169200.1
Gbar_A01	Gbar_A01G002810.1	==	D09	Gh_D09G257500.1
Gbar_A01	Gbar_A01G006610.1	==	D10	Gh_D10G016300.1
Gbar_A05	Gbar_A05G023670.1	==	A05	Gh_A05G223800.1
Gbar_A05	Gbar_A05G023670.1	==	D05	Gh_D05G240000.1
Gbar_A06	Gbar_A06G020170.1	==	A06	Gh_A06G217500.1
Gbar_A06	Gbar_A06G021100.1	==	A06	Gh_A06G227200.1
Gbar_A06	Gbar_A06G021100.1	==	A06	Gh_A06G161500.1
Gbar_A06	Gbar_A06G020170.1	==	A10	Gh_A10G066700.1
Gbar_A06	Gbar_A06G021100.1	==	A10	Gh_A10G033300.1
Gbar_A06	Gbar_A06G020170.1	==	D06	Gh_D06G223900.1
Gbar_A06	Gbar_A06G021100.1	==	D06	Gh_D06G234500.1
Gbar_A06	Gbar_A06G021100.1	==	D06	Gh_D06G162500.1
Gbar_A06	Gbar_A06G020170.1	==	D10	Gh_D10G074400.1
Gbar_A06	Gbar_A06G021100.1	==	D10	Gh_D10G042100.1
Gbar_A06	Gbar_A06G021100.1	==	D13	Gh_D13G174400.1
Gbar_A07	Gbar_A07G004340.1	==	A07	Gh_A07G044700.1
Gbar_A07	Gbar_A07G004340.1	==	D07	Gh_D07G045600.1
Gbar_A08	Gbar_A08G026180.1	==	A08	Gh_A08G275600.1
Gbar_A08	Gbar_A08G026180.1	==	D08	Gh_D08G266000.1
Gbar_A09	Gbar_A09G018990.1	==	A09	Gh_A09G196600.1
Gbar_A09	Gbar_A09G018990.1	==	D09	Gh_D09G051300.1
Gbar_A10	Gbar_A10G004440.1	==	A06	Gh_A06G161500.1
Gbar_A10	Gbar_A10G008380.1	==	A06	Gh_A06G217500.1
Gbar_A10	Gbar_A10G004440.1	==	A06	Gh_A06G227200.1
Gbar_A10	Gbar_A10G004440.1	==	A09	Gh_A09G094600.1
Gbar_A10	Gbar_A10G004440.1	==	A10	Gh_A10G033300.1
Gbar_A10	Gbar_A10G008380.1	==	A10	Gh_A10G066700.1
Gbar_A10	Gbar_A10G004440.1	==	D06	Gh_D06G162500.1
Gbar_A10	Gbar_A10G008380.1	==	D06	Gh_D06G223900.1
Gbar_A10	Gbar_A10G004440.1	==	D06	Gh_D06G234500.1
Gbar_A10	Gbar_A10G004440.1	==	D09	Gh_D09G089800.1
Gbar_A10	Gbar_A10G004440.1	==	D10	Gh_D10G042100.1
Gbar_A10	Gbar_A10G008380.1	==	D10	Gh_D10G074400.1
Gbar_A13	Gbar_A13G016640.1	==	A01	Gh_A01G038100.1
Gbar_A13	Gbar_A13G016640.1	==	A13	Gh_A13G172500.1
Gbar_A13	Gbar_A13G016640.1	==	D01	Gh_D01G035600.1
Gbar_A13	Gbar_A13G016640.1	==	D13	Gh_D13G174400.1
Gbar_D01	Gbar_D01G002990.1	==	A01	Gh_A01G032000.1
Gbar_D01	Gbar_D01G002990.1	==	A09	Gh_A09G268400.1
Gbar_D01	Gbar_D01G002990.1	==	D01	Gh_D01G029400.1

Gbar_D01	Gbar_D01G002990.1	==	D09	Gh_D09G257500.1
Gbar_D05	Gbar_D05G017240.1	==	A01	Gh_A01G076300.1
Gbar_D05	Gbar_D05G024240.1	==	A05	Gh_A05G223800.1
Gbar_D05	Gbar_D05G017240.1	==	A10	Gh_A10G006100.1
Gbar_D05	Gbar_D05G017240.1	==	D01	Gh_D01G071400.1
Gbar_D05	Gbar_D05G017240.1	==	D05	Gh_D05G169200.1
Gbar_D05	Gbar_D05G024240.1	==	D05	Gh_D05G240000.1
Gbar_D05	Gbar_D05G017240.1	==	D10	Gh_D10G016300.1
Gbar_D06	Gbar_D06G015470.1	==	A06	Gh_A06G161500.1
Gbar_D06	Gbar_D06G020970.1	==	A06	Gh_A06G217500.1
Gbar_D06	Gbar_D06G021870.1	==	A06	Gh_A06G227200.1
Gbar_D06	Gbar_D06G015470.1	==	A06	Gh_A06G227200.1
Gbar_D06	Gbar_D06G021870.1	==	A06	Gh_A06G161500.1
Gbar_D06	Gbar_D06G015470.1	==	A09	Gh_A09G094600.1
Gbar_D06	Gbar_D06G015470.1	==	A10	Gh_A10G033300.1
Gbar_D06	Gbar_D06G020970.1	==	A10	Gh_A10G066700.1
Gbar_D06	Gbar_D06G021870.1	==	A10	Gh_A10G033300.1
Gbar_D06	Gbar_D06G015470.1	==	D06	Gh_D06G162500.1
Gbar_D06	Gbar_D06G020970.1	==	D06	Gh_D06G223900.1
Gbar_D06	Gbar_D06G021870.1	==	D06	Gh_D06G234500.1
Gbar_D06	Gbar_D06G015470.1	==	D06	Gh_D06G234500.1
Gbar_D06	Gbar_D06G021870.1	==	D06	Gh_D06G162500.1
Gbar_D06	Gbar_D06G015470.1	==	D09	Gh_D09G089800.1
Gbar_D06	Gbar_D06G015470.1	==	D10	Gh_D10G042100.1
Gbar_D06	Gbar_D06G020970.1	==	D10	Gh_D10G074400.1
Gbar_D06	Gbar_D06G021870.1	==	D10	Gh_D10G042100.1
Gbar_D07	Gbar_D07G004640.1	==	A07	Gh_A07G044700.1
Gbar_D07	Gbar_D07G014160.1	==	A07	Gh_A07G139000.1
Gbar_D07	Gbar_D07G014160.1	==	A08	Gh_A08G046300.1
Gbar_D07	Gbar_D07G014160.1	==	A12	Gh_A12G140400.1
Gbar_D07	Gbar_D07G004640.1	==	D07	Gh_D07G045600.1
Gbar_D07	Gbar_D07G014160.1	==	D07	Gh_D07G138400.1
Gbar_D07	Gbar_D07G014160.1	==	D12	Gh_D12G140300.1
Gbar_D09	Gbar_D09G008430.1	==	A06	Gh_A06G161500.1
Gbar_D09	Gbar_D09G018790.1	==	A09	Gh_A09G196600.1
Gbar_D09	Gbar_D09G008430.1	==	A09	Gh_A09G094600.1
Gbar_D09	Gbar_D09G008430.1	==	A10	Gh_A10G033300.1
Gbar_D09	Gbar_D09G008430.1	==	D06	Gh_D06G162500.1
Gbar_D09	Gbar_D09G008430.1	==	D09	Gh_D09G089800.1
Gbar_D09	Gbar_D09G018790.1	==	D09	Gh_D09G051300.1
Gbar_D09	Gbar_D09G008430.1	==	D10	Gh_D10G042100.1
Gbar_D10	Gbar_D10G004310.1	==	A06	Gh_A06G161500.1
Gbar_D10	Gbar_D10G007440.1	==	A06	Gh_A06G217500.1
Gbar_D10	Gbar_D10G004310.1	==	A06	Gh_A06G227200.1
Gbar_D10	Gbar_D10G004310.1	==	A09	Gh_A09G094600.1
Gbar_D10	Gbar_D10G004310.1	==	A10	Gh_A10G033300.1
Gbar_D10	Gbar_D10G007440.1	==	A10	Gh_A10G066700.1
Gbar_D10	Gbar_D10G004310.1	==	D06	Gh_D06G162500.1
Gbar_D10	Gbar_D10G007440.1	==	D06	Gh_D06G223900.1
Gbar_D10	Gbar_D10G004310.1	==	D06	Gh_D06G234500.1

Gbar_D10	Gbar_D10G004310.1	==	D09	Gh_D09G089800.1
Gbar_D10	Gbar_D10G004310.1	==	D10	Gh_D10G042100.1
Gbar_D10	Gbar_D10G007440.1	==	D10	Gh_D10G074400.1
Gbar_D13	Gbar_D13G016610.1	==	A01	Gh_A01G038100.1
Gbar_D13	Gbar_D13G016610.1	==	A13	Gh_A13G172500.1
Gbar_D13	Gbar_D13G016610.1	==	D01	Gh_D01G035600.1
Gbar_D13	Gbar_D13G016610.1	==	D13	Gh_D13G174400.1
Gbar_A01	Gbar_A01G002810.1	==	Chr01	Grai_01G003210.1
Gbar_A01	Gbar_A01G006610.1	==	Chr01	Grai_01G007910.1
Gbar_A01	Gbar_A01G006610.1	==	Chr05	Grai_05G017890.1
Gbar_A01	Gbar_A01G006610.1	==	Chr10	Grai_10G001630.1
Gbar_A05	Gbar_A05G023670.1	==	Chr05	Grai_05G025290.1
Gbar_A06	Gbar_A06G020170.1	==	Chr06	Grai_06G025490.1
Gbar_A06	Gbar_A06G021100.1	==	Chr06	Grai_06G026430.1
Gbar_A06	Gbar_A06G021100.1	==	Chr06	Grai_06G018730.1
Gbar_A06	Gbar_A06G020170.1	==	Chr10	Grai_10G007910.1
Gbar_A06	Gbar_A06G021100.1	==	Chr10	Grai_10G004350.1
Gbar_A07	Gbar_A07G004340.1	==	Chr07	Grai_07G024570.1
Gbar_A08	Gbar_A08G026180.1	==	Chr08	Grai_08G031240.1
Gbar_A09	Gbar_A09G018990.1	==	Chr09	Grai_09G021550.1
Gbar_A09	Gbar_A09G018990.1	==	Chr09	Grai_09G006000.1
Gbar_A10	Gbar_A10G004440.1	==	Chr06	Grai_06G018730.1
Gbar_A10	Gbar_A10G008380.1	==	Chr06	Grai_06G025490.1
Gbar_A10	Gbar_A10G004440.1	==	Chr06	Grai_06G026430.1
Gbar_A10	Gbar_A10G004440.1	==	Chr09	Grai_09G010700.1
Gbar_A10	Gbar_A10G004440.1	==	Chr10	Grai_10G004350.1
Gbar_A10	Gbar_A10G008380.1	==	Chr10	Grai_10G007910.1
Gbar_A13	Gbar_A13G016640.1	==	Chr01	Grai_01G003860.1
Gbar_A13	Gbar_A13G016640.1	==	Chr13	Grai_13G020810.1
Gbar_D01	Gbar_D01G002990.1	==	Chr01	Grai_01G003210.1
Gbar_D01	Gbar_D01G002990.1	==	Chr09	Grai_09G028620.1
Gbar_D05	Gbar_D05G017240.1	==	Chr01	Grai_01G007910.1
Gbar_D05	Gbar_D05G017240.1	==	Chr05	Grai_05G017890.1
Gbar_D05	Gbar_D05G024240.1	==	Chr05	Grai_05G025290.1
Gbar_D05	Gbar_D05G017240.1	==	Chr10	Grai_10G001630.1
Gbar_D06	Gbar_D06G015470.1	==	Chr06	Grai_06G018730.1
Gbar_D06	Gbar_D06G020970.1	==	Chr06	Grai_06G025490.1
Gbar_D06	Gbar_D06G021870.1	==	Chr06	Grai_06G026430.1
Gbar_D06	Gbar_D06G015470.1	==	Chr06	Grai_06G026430.1
Gbar_D06	Gbar_D06G021870.1	==	Chr06	Grai_06G018730.1
Gbar_D06	Gbar_D06G015470.1	==	Chr09	Grai_09G010700.1
Gbar_D06	Gbar_D06G015470.1	==	Chr10	Grai_10G004350.1
Gbar_D06	Gbar_D06G020970.1	==	Chr10	Grai_10G007910.1
Gbar_D06	Gbar_D06G021870.1	==	Chr10	Grai_10G004350.1
Gbar_D07	Gbar_D07G014160.1	==	Chr07	Grai_07G014260.1
Gbar_D07	Gbar_D07G004640.1	==	Chr07	Grai_07G024570.1
Gbar_D07	Gbar_D07G014160.1	==	Chr08	Grai_08G004830.1
Gbar_D07	Gbar_D07G014160.1	==	Chr12	Grai_12G016030.1
Gbar_D09	Gbar_D09G008430.1	==	Chr06	Grai_06G018730.1
Gbar_D09	Gbar_D09G008430.1	==	Chr09	Grai_09G010700.1

Gbar_D09	Gbar_D09G018790.1	==	Chr09	Grai_09G021550.1
Gbar_D09	Gbar_D09G018790.1	==	Chr09	Grai_09G006000.1
Gbar_D09	Gbar_D09G008430.1	==	Chr10	Grai_10G004350.1
Gbar_D10	Gbar_D10G004310.1	==	Chr06	Grai_06G018730.1
Gbar_D10	Gbar_D10G007440.1	==	Chr06	Grai_06G025490.1
Gbar_D10	Gbar_D10G004310.1	==	Chr06	Grai_06G026430.1
Gbar_D10	Gbar_D10G004310.1	==	Chr09	Grai_09G010700.1
Gbar_D10	Gbar_D10G004310.1	==	Chr10	Grai_10G004350.1
Gbar_D10	Gbar_D10G007440.1	==	Chr10	Grai_10G007910.1
Gbar_D13	Gbar_D13G016610.1	==	Chr01	Grai_01G003860.1
Gbar_D13	Gbar_D13G016610.1	==	Chr13	Grai_13G020810.1
Gbar_A01	Gbar_A01G002810.1	==	Chr01	Ga01G0322
Gbar_A01	Gbar_A01G006610.1	==	Chr01	Ga01G0804
Gbar_A01	Gbar_A01G006610.1	==	Chr05	Ga05G1796
Gbar_A01	Gbar_A01G006610.1	==	Chr10	Ga10G2934
Gbar_A05	Gbar_A05G023670.1	==	Chr05	Ga05G2543
Gbar_A06	Gbar_A06G020170.1	==	Chr06	Ga06G2297
Gbar_A06	Gbar_A06G021100.1	==	Chr06	Ga06G2463
Gbar_A06	Gbar_A06G021100.1	==	Chr06	Ga06G1671
Gbar_A06	Gbar_A06G021100.1	==	Chr10	Ga10G2652
Gbar_A06	Gbar_A06G020170.1	==	Chr10	Ga10G2260
Gbar_A07	Gbar_A07G004340.1	==	Chr07	Ga07G0488
Gbar_A08	Gbar_A08G026180.1	==	Chr08	Ga08G2824
Gbar_A09	Gbar_A09G018990.1	==	Chr09	Ga09G1936
Gbar_A10	Gbar_A10G004440.1	==	Chr06	Ga06G1671
Gbar_A10	Gbar_A10G004440.1	==	Chr06	Ga06G2463
Gbar_A10	Gbar_A10G008380.1	==	Chr06	Ga06G2297
Gbar_A10	Gbar_A10G004440.1	==	Chr09	Ga09G0935
Gbar_A10	Gbar_A10G004440.1	==	Chr10	Ga10G2652
Gbar_A10	Gbar_A10G008380.1	==	Chr10	Ga10G2260
Gbar_A13	Gbar_A13G016640.1	==	Chr01	Ga01G0389
Gbar_A13	Gbar_A13G016640.1	==	Chr13	Ga13G1903
Gbar_D01	Gbar_D01G002990.1	==	Chr01	Ga01G0322
Gbar_D01	Gbar_D01G002990.1	==	Chr09	Ga09G2749
Gbar_D05	Gbar_D05G017240.1	==	Chr01	Ga01G0804
Gbar_D05	Gbar_D05G017240.1	==	Chr05	Ga05G1796
Gbar_D05	Gbar_D05G024240.1	==	Chr05	Ga05G2543
Gbar_D05	Gbar_D05G017240.1	==	Chr10	Ga10G2934
Gbar_D06	Gbar_D06G015470.1	==	Chr06	Ga06G1671
Gbar_D06	Gbar_D06G020970.1	==	Chr06	Ga06G2297
Gbar_D06	Gbar_D06G015470.1	==	Chr06	Ga06G2463
Gbar_D06	Gbar_D06G021870.1	==	Chr06	Ga06G2463
Gbar_D06	Gbar_D06G021870.1	==	Chr06	Ga06G1671
Gbar_D06	Gbar_D06G015470.1	==	Chr09	Ga09G0935
Gbar_D06	Gbar_D06G021870.1	==	Chr10	Ga10G2652
Gbar_D06	Gbar_D06G015470.1	==	Chr10	Ga10G2652
Gbar_D06	Gbar_D06G020970.1	==	Chr10	Ga10G2260
Gbar_D07	Gbar_D07G004640.1	==	Chr07	Ga07G0488
Gbar_D07	Gbar_D07G014160.1	==	Chr07	Ga07G1447
Gbar_D07	Gbar_D07G014160.1	==	Chr08	Ga08G0461

Gbar_D07	Gbar_D07G014160.1	==	Chr12	Ga12G1673
Gbar_D09	Gbar_D09G008430.1	==	Chr06	Ga06G1671
Gbar_D09	Gbar_D09G008430.1	==	Chr09	Ga09G0935
Gbar_D09	Gbar_D09G018790.1	==	Chr09	Ga09G1936
Gbar_D09	Gbar_D09G008430.1	==	Chr10	Ga10G2652
Gbar_D10	Gbar_D10G004310.1	==	Chr06	Ga06G1671
Gbar_D10	Gbar_D10G004310.1	==	Chr06	Ga06G2463
Gbar_D10	Gbar_D10G007440.1	==	Chr06	Ga06G2297
Gbar_D10	Gbar_D10G004310.1	==	Chr09	Ga09G0935
Gbar_D10	Gbar_D10G007440.1	==	Chr10	Ga10G2260
Gbar_D10	Gbar_D10G004310.1	==	Chr10	Ga10G2652
Gbar_D13	Gbar_D13G016610.1	==	Chr01	Ga01G0389
Gbar_D13	Gbar_D13G016610.1	==	Chr13	Ga13G1903

Table S4. Primer sequence used for qRT-PCR.

Gene name	Upstream primer	Downstream primer
Q7	GAAGGCATTCCACCTGACCAAC	CTTGACCTTCTTCTTCTTGTGCTTG
GA2ox2f	AATGGGAGGCTAAGAAGTGTAAGG	GCCTATTAGCACCCAACCTTGAA
GA20ox1	CCAAGTACCACTCATTGACCTTG	GAAAGAAACCATGCCGTTGG
Gbar_D02G010640	GTGCAAATCGAATACTTCACCG	AGTTCTTTTGTACCCACTGCAATC
Gbar_A05G000630	TACCTTGCTTGTTCCGCTTATG	AAACCAGAACCAGAAGCTTTATCA
Gbar_D05G000620	TTTACCTTGCTTGTTCCTCTTATTG	GACGATTTTACCACGGCCA
Gbar_D03G005050	GTGATCCTTACATTCTTCACACAAAT	TACTTTGGGGGTTCCAGAGG
Gbar_A02G015220	TGACCCTTACATTCTTCACACAAAC	GGAACATAACAATATCAAATACTCCG
Gbar_D08G002810	CATTGCTGCTACTATCTGCCTTAA	CGAGGGTGAGAGTGAGAAGGTT
Gbar_A08G002700	GATGTTACTTGGGGTGATGGC	ACAGTGCCAGCAGAGTTGCC
GA2ox1a	CAGCTACATTGTTCCCTCCG	TGGTGGCTTCAGATTCCAGC
GA2ox1b	CAGCTACATTGTTCCCTCCT	TGGTGGCTTCAGATTCTATG
GA20ox2a	TGATTGCGTTTCCAAGATACCT	GCATTAACATTAGGCTTTTCGTG
